# Transcriptional modulation of the PI3K/AKT/mTOR signaling pathway mediated by HPV16 *E5*, *E6*, and *E7* oncogene expression in breast cancer

**DOI:** 10.37349/etat.2026.1002391

**Published:** 2026-08-04

**Authors:** Beatriz Eda de Oliveira Isídio, Pedro Henrique Bezerra Fontes, Gabriel Rômulo Parente da Silva, Stephanie Loureiro Leão, Bianca de França São Marcos, Isabelle Silva Simões, Elisa Fotin Genn Barros, David Beltrán Lussón, Gabriela Vitória de Araujo, Isabela Duarte de Farias, Karina Mayumi Tani Bezerra de Melo, Nathálya Lima de Queiroz, Sandra Maria Souza da Silva, Vanessa Emanuelle Pereira Santos, Antonio Carlos de Freitas

**Affiliations:** IRCCS Istituto Romagnolo per lo Studio dei Tumori (IRST) “Dino Amadori”, Italy; Laboratory of Molecular Studies and Experimental Therapy, Department of Genetics, Federal University of Pernambuco, Recife 50670-901, PE, Brazil

**Keywords:** human papillomavirus, breast cancer carcinogenesis, PI3K/AKT/mTOR pathway

## Abstract

**Aim:**

Breast cancer is the most prevalent malignant tumor among women. Human papillomavirus (HPV) has been detected in breast tumors since the 1990s, and beyond its oncogenic potential, therapy resistance driven by viral immune evasion in non-anogenital tumors, such as oropharyngeal cancers, highlights the need to investigate viral activity in breast tissues. Among high-risk HPV types, HPV16 is one of the most prevalent and exhibits the highest carcinogenic potential. Therefore, this study aimed to evaluate the expression of HPV16 oncogenes *E5*, *E6*, and *E7* in breast tumors, as well as the modulation of the PI3K/AKT/mTOR signaling pathway associated with viral activity.

**Methods:**

A total of 92 breast cancer patients were included after Ethics Committee approval. Clinical data were obtained from medical records. RNA was extracted from formalin-fixed, paraffin-embedded tissues and reverse-transcribed into cDNA. Transcripts of HPV oncogenes (*E5*, *E6*, and *E7*), components of the PI3K/AKT/mTOR pathway, and regulatory genes (*EGFR* and *PTEN*) were quantified by RT-qPCR. Gene expression levels were calculated using the ΔCt method.

**Results:**

Forty-eight samples met RNA quality criteria and were included in the expression analysis. Among these, 77.08% showed expression of at least one viral oncogene, with *E5* being the most frequently expressed. The PI3K/AKT/mTOR pathway was modulated in HPV-positive samples, with increased PI3K expression and decreased mTOR expression. Notably, the high expression of E5—associated with immune evasion—combined with reduced mTOR expression suggests that HPV16 status may influence therapeutic response in breast cancer patients.

**Conclusions:**

These findings reinforce the importance of further studies investigating HPV activity in breast tumors to better understand its biological and clinical impact.

## Graphical abstract

**Graphical abstract.**
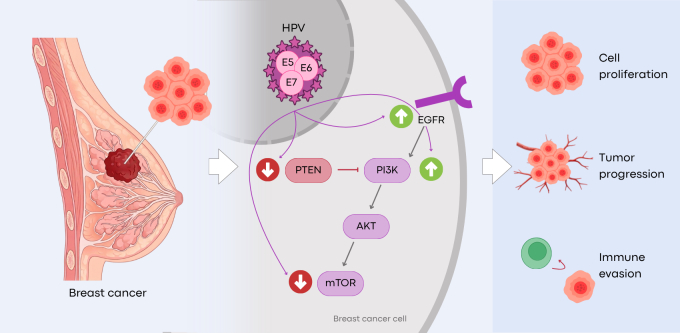
HPV-mediated alterations in the PI3K/AKT/mTOR pathway and its regulators EGFR and PTEN.

## Introduction

Breast cancer is the leading cause of cancer-related mortality among women worldwide and the most prevalent malignancy in this population [1]. In 2022, approximately 2.3 million new cases were identified, according to data from the Global Cancer Observatory (GLOBOCAN) of the International Agency for Research on Cancer (IARC) [2]. The main risk factors for the development of breast cancer include a family history of the disease, mutations in the *BRCA1* and *BRCA2* genes, prolonged use of hormone therapy, and lifestyle factors [3]. In addition, several viral infections have been investigated as potential contributors to breast carcinogenesis, including Epstein–Barr virus (EBV), mouse mammary tumor virus (MMTV), and human papillomavirus (HPV) [3].

HPV is a double-stranded DNA virus comprising more than 200 types, which are classified as low- or high-risk according to their oncogenic potential [4]. Among the high-risk types, HPV16 is the most prevalent, accounting for approximately 89% of HPV-positive oropharyngeal cancers and 52% of cervical cancer cases [5–7]. Furthermore, most lesions classified as grade 3 epithelial neoplasia (CIN3), considered premalignant and more likely to progress to invasive cancer, are associated with infection by this viral genotype [5, 7, 8].

The first evidence of an association between HPV and breast cancer was reported in 1992, and since then, numerous studies have explored this relationship [9, 10]. Studies assessing HPV prevalence in breast tissue report a wide variation ranging from 0 to 86.2%, with a pooled prevalence of 23% for viral DNA positivity, with genotype 16 being the most prevalent in these tissues [11, 12]. More recently, a study by Sakhi et al. [13] detected HPV in breast carcinoma tissues, of which 65.4% were positive for HPV16. However, other studies have not detected the presence of HPV16 in breast cancer tissues, highlighting the variability in HPV detection rates and viral genotype distribution among different studies [13–16]. Women with HPV-positive tumors have an approximately 5.9-fold higher likelihood of presenting a worse prognosis in invasive ductal carcinoma compared with those with HPV-negative tumors [17]. Additionally, the presence of HPV in breast tissue has also been associated with more aggressive grade III tumors and high expression of Ki-67, a protein that reflects the tumor cell proliferation rate [13].

HPV can modulate multiple signaling pathways involved in inflammation, cell proliferation, and immune response [18]. This modulation constitutes a viral mechanism that contributes to the initiation of carcinogenesis [19]. In studies of HPV-positive head and neck squamous cell carcinoma, increased activation of the PI3K/AKT/mTOR pathway has been observed. This pathway regulates cell proliferation, survival, invasion, migration, apoptosis, glucose metabolism, and DNA repair, and its activation is higher in HPV-positive cases than in HPV-negative ones [20].

This pathway is activated by the epidermal growth factor receptor (EGFR), a transmembrane glycoprotein and member of the protein kinase superfamily [21, 22]. EGFR is a cell surface receptor that binds epidermal growth factor, triggering receptor dimerization and tyrosine autophosphorylation, ultimately promoting cell proliferation [22, 23]. Studies have shown that elevated EGFR expression is associated with poor prognosis in breast cancer, particularly in triple-negative and HER2-positive subtypes [24, 25]. The negative regulator of this pathway, phosphatase and tensin homolog (PTEN), functions as a phosphatase by dephosphorylating phosphatidylinositol (3,4,5)-trisphosphate (PIP3) to phosphatidylinositol 4,5-bisphosphate (PIP2), thereby interrupting the signal that activates the serine/threonine kinase AKT [20, 26]. In the absence of PTEN, AKT and the mammalian target of rapamycin (mTOR) become hyperactivated, leading to uncontrolled cell growth and resistance to apoptosis [20].

The PI3K/AKT/mTOR pathway plays a central role in breast cancer by regulating cell growth, survival, and tumor progression. Consequently, it has emerged as a key therapeutic target in the management of this malignancy [27–29]. Inhibitors of PI3K, AKT, and mTOR have been investigated in the treatment of different molecular subtypes of breast cancer, with some already approved by the Food and Drug Administration (FDA), such as everolimus, an mTOR inhibitor, and alpelisib, a PI3K inhibitor, indicated for luminal-type cancers [30]. In this context, investigating how HPV influences this pathway—particularly through its oncoproteins E5, E6, and E7—may reveal mechanisms by which the virus contributes to breast carcinogenesis and help identify novel therapeutic approaches for HPV-associated breast neoplasms. Accordingly, the aim of the present study is to elucidate the role of HPV in modulating the PI3K/AKT/mTOR pathway in breast tumors, thereby contributing to a better understanding of the relationship between HPV and breast cancer.

## Materials and methods

### Biological samples

This study evaluated patients with a confirmed diagnosis of breast cancer who were treated at the Hospital das Clínicas of the Federal University of Pernambuco (HC-UFPE), located in Recife, Pernambuco, Brazil. Patients with primary breast tumors, aged over 18 years, and those who provided written informed consent were included. Patients who did not meet the inclusion criteria, as well as those with cervical and/or head and neck cancers, were excluded. Formalin-fixed, paraffin-embedded (FFPE) breast tumor samples from each patient were collected between June 2019 and December 2023. Clinical data were obtained from medical records, including age, histological type, and molecular subtype, which were recorded in spreadsheets and subsequently analyzed. This study was approved by the Research Ethics Committee of the Federal University of Pernambuco, Brazil, under protocol number CAAE 40062720.0.0000.5208.

### RNA extraction and cDNA synthesis

RNA was extracted using the TRIzol/chloroform method, following the manufacturer’s instructions with minor modifications. Briefly, tumor tissues were sectioned and weighed, totaling 150 mg of material per sample. TRIzol and chloroform were added at a 5:1 ratio, followed by centrifugation at 4°C and precipitation at –80°C for 20 minutes. After this step, samples were centrifuged again to precipitate the pellet, which was then purified using 75% ethanol. Finally, the pellet was resuspended in nuclease-free water, and 1.0 μL of DNase was added and incubated at 37°C for 15 minutes. RNA was also quantified using a NanoDrop (Thermo Scientific^®^, Waltham, MA, USA), and only samples with RNA concentrations above 125 ng/µL and 260/280 ratios within the range of 1.8–2.2 were selected (Table S1). Following extraction, total RNA was used for cDNA synthesis using the Applied Biosystems High-Capacity cDNA Reverse Transcription Kit (Thermo Scientific^®^, Waltham, MA, USA), for subsequent real-time quantitative PCR (RT-qPCR) analysis.

### Relative expression analysis of viral oncogenes and the PI3K/AKT/mTOR pathway via RT-qPCR

Expression analysis was performed for the HPV16 oncogenes as well as key genes of the PI3K/AKT/mTOR pathway, including its main activator and inhibitor. To ensure efficient amplification from FFPE tissues—which are often subject to nucleic acid degradation due to the paraffinization process [31, 32]—a preliminary conventional PCR was performed for the HPV16 oncogenes *E5*, *E6*, and *E7*, as well as for pathway-related genes, including *PI3K*, *AKT*, *mTOR*, *PTEN*, and *EGFR*. The same approach was applied to the reference genes (endogenous) used for normalization in this study, *GAPDH* and *EEF1A1*. In addition to the reference genes, an HPV-positive cervical cancer sample, extracted by the same process described above, was also used as a reference to obtain the relative expression of HPV oncogene mRNAs. Target gene amplification was conducted using 96-well plates on a LineGene 9660 thermocycler (Bioer Technology, Hangzhou, Zhejiang, China), following the SYBR Green detection method. All reactions were performed in accordance with MIQE guidelines [33], with two technical replicates for each experimental condition. The RT-qPCR cycling conditions were: 95°C for 2 minutes, followed by 40 cycles of 95°C for 15 seconds and 60°C for 1 minute. Primers for the target genes were selected based on the literature and are listed below; primer efficiency values and melting curve analyses are provided in Tables S1, Table S2, and Figure S1. Relative gene expression was calculated using ΔCt (Ct gene – Ct endogenous) [34]. For ease of interpretation, –ΔCt values were used, where higher values indicate greater relative expression. Additionally, effect size and post-hoc analyses were performed to ensure the robustness of the results. All analyses were conducted using GraphPad Prism software (version 9.0.0; GraphPad Software Inc., San Diego, CA, USA) and RStudio (version 4.5.3; Posit Software, PBC, Boston, MA, USA).

### Statistical analysis

Statistical analyses were performed using −ΔCt values. Comparisons between two groups were conducted using the Mann–Whitney *U* test, while comparisons among multiple groups were performed using the Kruskal–Wallis test. Gene–gene correlations were assessed using Spearman’s rank correlation coefficient. Results with *p*-values < 0.05 were considered statistically significant. For comparisons between HPV16-positive and HPV16-negative groups, odds ratios (ORs) and their corresponding *p*-values were calculated, considering variables such as age, presence of metastasis, and tumor molecular subtype. Correlation analyses were performed using Spearman’s rank correlation test in GraphPad Prism (version 9.0.0; GraphPad Software, Inc., San Diego, CA, USA), which is appropriate for non-normally distributed data. Principal component analysis (PCA) was conducted using standardized data (*z*-score scaling), and clustering was performed using the *k*-means algorithm with silhouette validation. All graphical representations (boxplots, PCA plots, and heatmaps) were generated based on −ΔCt values. PCA was additionally performed in RStudio (R Foundation for Statistical Computing, Vienna, Austria; version 2024.12.1) using the packages dplyr, ggplot2, and factoextra.

## Results

### Expression of HPV16 oncogenes in breast tumor samples and patient characterization

Based on the inclusion and exclusion criteria, 92 samples were collected; however, high-quality RNA (based on concentration in ng, 260/280 ratio, and successful amplification of reference genes) was obtained from only 48 of them (47%) (Table S1). Samples with adequate RNA were characterized according to age, tumor subtype, and HPV status, defined as positive when expression of HPV16 oncogenes was detected and negative when no expression was identified (Table 1). Among the 48 samples, 37 (77.08%) were HPV16-positive, whereas 11 (22.91%) were negative. The cohort predominantly comprised patients aged 51–70 years, regardless of HPV status. Within the HPV-positive group, the most prevalent molecular subtypes were luminal A and luminal B (Table 1).

**Table 1:** Characterization of the samples regarding patient age, tumor type, and expression of HPV16 oncogenes in the samples.

**Clinical and histopathological characteristics**	**HPV16-positive**	**HPV16-negative**	**OR**	**IC_50_**	***p*-value**
**Age**			1.439		*p* global (0.7714)
30–50 (ref)	10	2	1.00		
51–70	17	7	0.486	0.084–2.809	0.4199
> 70	8	2	0.800	0.091–7.002	0.8402
Not-identified	2	0	1.190	0.042–33.427	0.9184
**Total**	37	11			
**Molecular type**					*p* global (0.6296)
HER-2	1	1	0.235	0.012–4.624	0.3410
Luminal A (ref)	17	4	1.00		
Luminal B	13	4	0.765	0.16–3.649	0.7365
Triple-negative	3	0	1.800	0.078–41.55	0.7136
Not-identified	3	2	0.353	0.043–2.867	0.3298
**Total**	37	11			
**Histological type**					*p* global (0.268)
Ductal in situ	5	1	1.111	0.109–11.331	0.9291
Invasive ductal (ref)	27	6	1.00		
Lobular in situ	0	0			
Invasive lobular	0	0			
Other types	1	2	0.111	0.009–1.435	0.0923
Not-identified	4	2	0.444	0.066–3.014	0.4063
**Total**	37	11			

The ages of the patients ranged from 30–50 years, 51–70 years, and over 70 years. Regarding HPV16 status, patients were classified as HPV16-positive or HPV16-negative. Concerning tumor subtype, patients were categorized as luminal A, luminal B, HER2-positive, or triple-negative. Odds ratios (ORs) and 95% confidence intervals (CIs) for the association between clinicopathological variables and HPV16 status. The reference categories were: age (30–50 years), molecular subtype (luminal A), and histological type (invasive ductal carcinoma).

### Relative expression of viral oncogenes *E5*, *E6*, and *E7*

**Figure 1:**
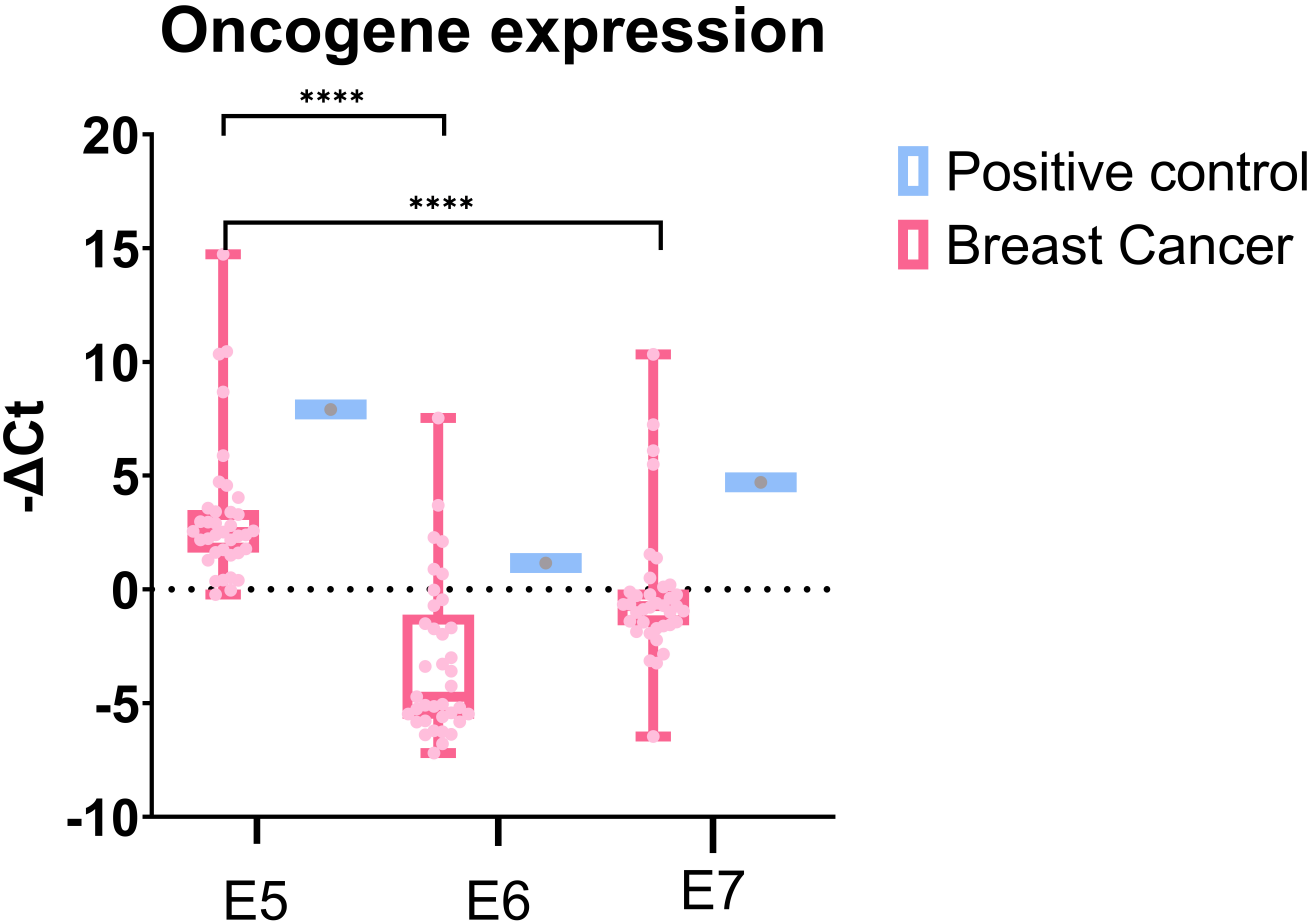
**Relative expression of the oncogenes *E5*, *E6*, and *E7* in HPV16-positive breast cancer patients in comparison to the cervical cancer sample (positive control).** A significant increase in *E5* expression was observed compared to E7 and E6 (^****^ *p* < 0.0001). No statistically significant difference was found between the expression levels of *E6* and *E7*. The analyses were evaluated using Dunn’s post-hoc test.

Moreover, to classify samples as HPV-positive or -negative based on oncogene expression, relative expression levels were compared among HPV-positive samples. This analysis showed that the *E5* oncogene exhibited higher expression than *E6* and *E7*, with *p*-values < 0.05 for E6 and < 0.001 for E7 (Figure 1, Table S2 and Figure S1). No significant difference was observed between the expression levels of *E6* and *E7*. This finding suggests higher activity of the *E5* oncogene relative to *E6* and *E7*, which are more commonly investigated and linked to carcinogenesis.

### Modulation of PI3K, AKT, and mTOR signaling and their regulatory genes by HPV16

**Figure 2:**
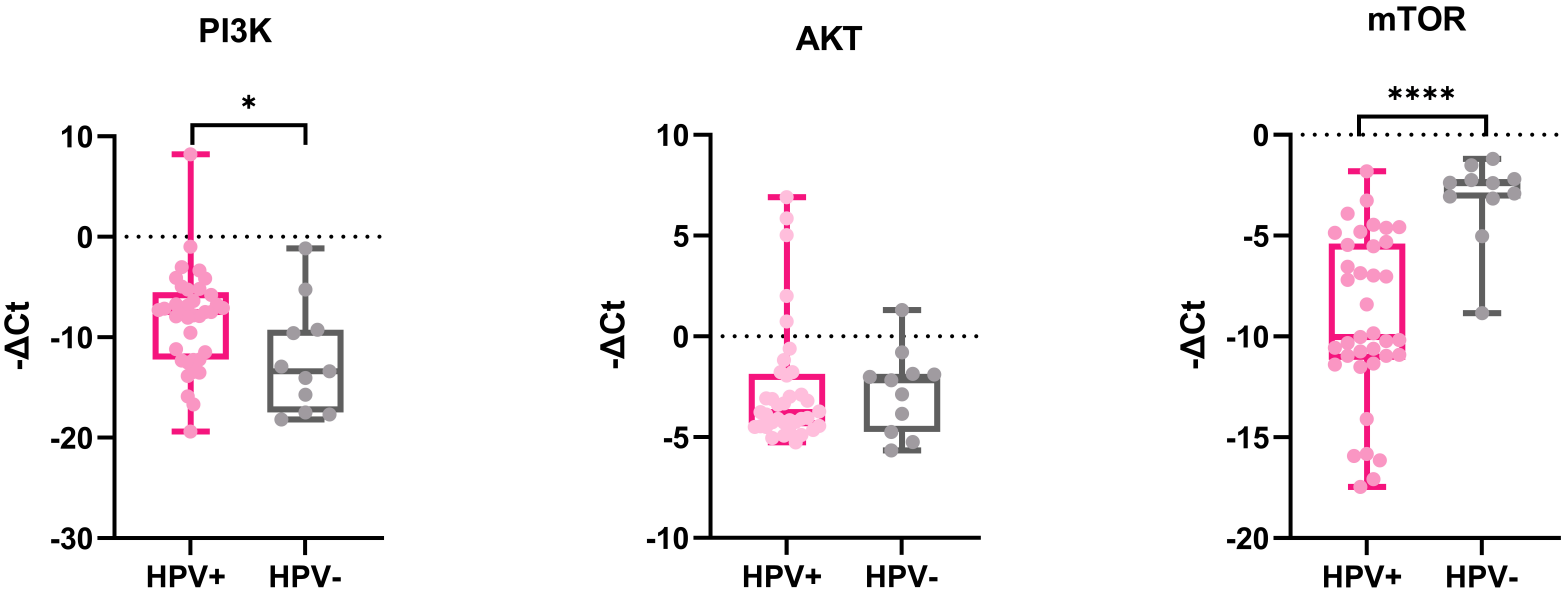
**Expression of *PI3K*, *AKT*, and *mTOR* genes in HPV16-positive and HPV16-negative breast cancer samples.** (**A**) It shows a significant increase in *PI3K* expression in the HPV16-positive group and a decrease in the HPV16-negative group (^*^ *p* < 0.05). (**B**) There is no significant difference in *AKT* expression between HPV16-positive and HPV16-negative groups. (**C**) It shows a significant decrease in *mTOR* expression in the HPV16-positive group and an increase in the HPV16-negative group (^****^ *p* < 0.0001).

The relative expression of genes associated with the PI3K/AKT/mTOR pathway was compared between HPV16-negative and HPV16-positive breast cancer groups (Table S2 and Figure S1). The HPV16-positive group exhibited significantly higher expression of PI3K (*p* < 0.05) and lower expression of mTOR (*p* < 0.001) (Figures 2A and C). However, no statistically significant difference in AKT expression levels was observed between the two groups (Figure 2B). These results suggest that pathway activation may involve a non-classical signaling cascade under certain conditions.

**Figure 3:**
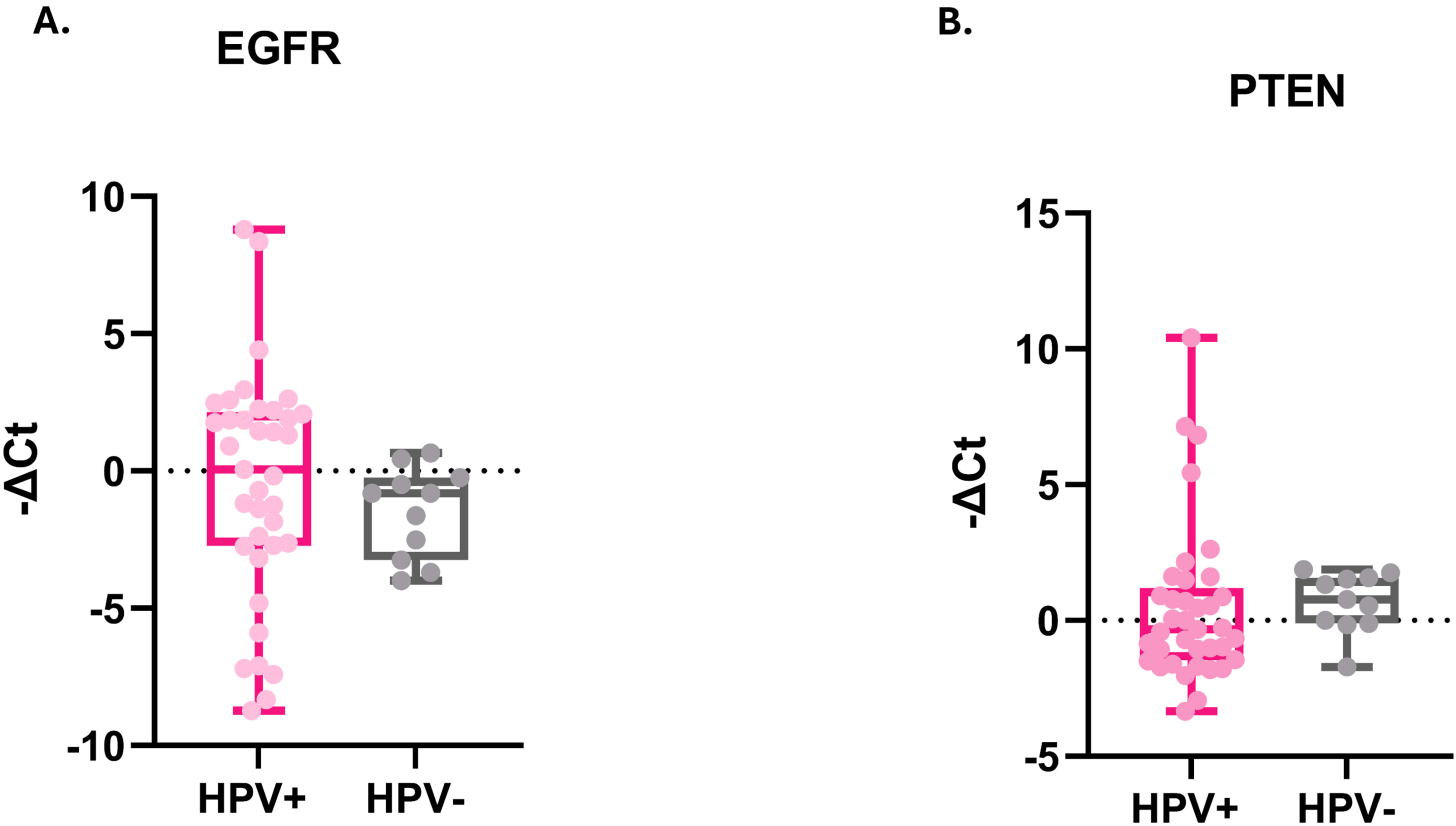
**Expression of *EGFR* and *PTEN* genes in HPV-positive and HPV16-negative breast cancer samples.** (**A**) There is no statistically significant difference in *EGFR* expression between HPV16-positive and HPV-negative groups. (**B**) It shows a trend toward increased *PTEN* expression in the HPV16-negative group; however, no statistically significant difference was observed between HPV16-positive and HPV-negative groups.

Relative expression analysis of regulatory genes within the PI3K/AKT/mTOR pathway showed no statistically significant difference in the *EGFR*, the primary activator of PI3K, between HPV16-positive and HPV16-negative breast cancer groups (Figure 3A, Table S2 and Figure S1). In addition, the negative regulator of this signaling pathway, *PTEN*, showed a trend toward increased expression in the HPV-negative group (Figure 3B).

To quantify the magnitude of differences between HPV-positive and HPV-negative groups, Cliff’s Delta was calculated (Table 2). The results showed that mTOR and PI3K exhibited the largest effect sizes, both classified as large (|δ| ≥ 0.43). Specifically, mTOR showed significantly lower expression in HPV-positive samples [δ = −0.843; 95% confidence interval (CI): −0.398 to 0.386; *p* < 0.001], whereas PI3K displayed significantly higher expression in HPV-positive samples (δ = 0.460; 95% CI: −0.391 to 0.401; *p* = 0.021). These findings indicate that HPV infection is associated with robust alterations in the expression of these two genes. *PTEN* showed a medium effect size (δ = −0.288; 95% CI: −0.388 to 0.388), with a non-significant trend toward lower expression in HPV-positive samples (*p* = 0.157). EGFR exhibited a small effect size (δ = 0.268; 95% CI: −0.393 to 0.398; *p* = 0.188), while AKT showed a negligible effect size (δ = −0.106; 95% CI: −0.388 to 0.383; *p* = 0.611).

**Table 2:** Cliff’s Delta analysis of PI3K/AKT/mTOR pathway regulation in HPV-positive and HPV-negative breast cancer.

**Gene**	**Median HPV+**	**Median HPV–**	***p*-value**	**Signif**	**Cliff’s Delta**	**95% CI**	**Effect**	**Direction**
*EGFR*	0.066	–0.803	0.188	ns	0.268	–0.0536 to 0.5389	Low	HPV+ > HPV–
*PI3K*	–7.488	–1.340	0.021	*	0.460	0.0014 to 0.7582	High	HPV+ >> HPV–
*AKT*	–3.761	–2.168	0.611	ns	–0.106	–0.4964 to 0.3207	Ineffective	HPV+ < HPV–
*PTEN*	–0.321	0.769	0.157	ns	–0.288	–0.5791 to 0.0694	Middle	HPV+ < HPV–
*mTOR*	–10.023	–2.394	< 0.001	***	–0.843	–0.9713 to –0.3327	High	HPV+ << HPV–

ns: not significant; CI: confidence interval. ^*^ *p*-value < 0.05; ^***^ *p*-value < 0.001.

### Correlation analysis

**Figure 4:**
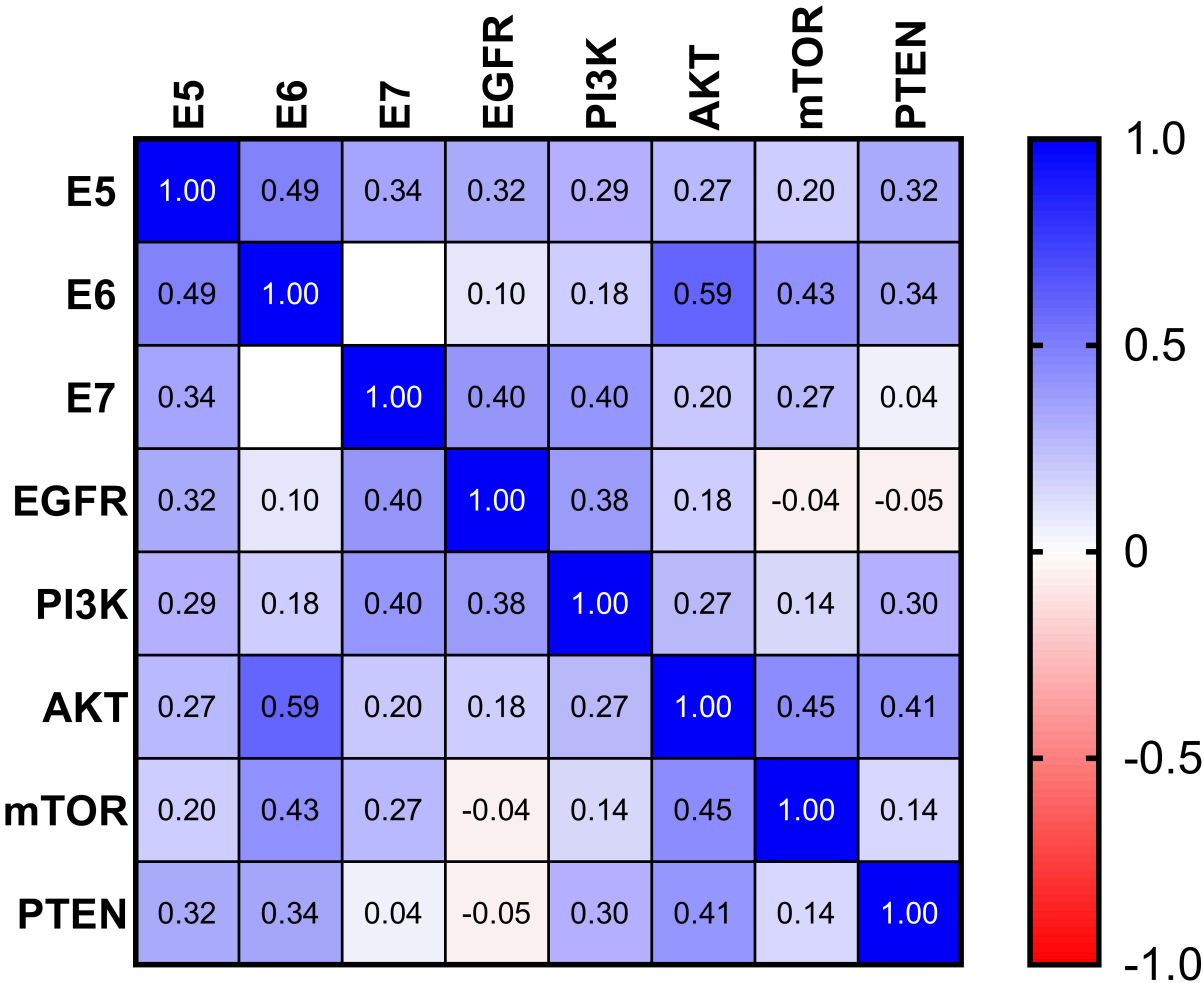
**Spearman correlation analysis was performed using GraphPad Prism.** Blue indicates a positive correlation between the genes evaluated, red indicates a negative correlation, and white indicates no correlation. A moderate positive correlation was found between E6 and AKT, and weak positive correlations were observed between E7 and EGFR, E7 and PI3K, AKT and mTOR, and EGFR and PI3K.

Spearman’s correlation analysis revealed statistically significant associations between viral oncogenes and components of the PI3K/AKT/mTOR pathway in the analyzed cancer samples. Notably, a moderate positive correlation was observed between E6 and AKT (*r* = 0.59; *p* = 0.0002), as well as weak positive correlations between E7 and EGFR (*r* = 0.40; *p* = 0.0186) and between E7 and PI3K (*r* = 0.40; *p* = 0.0194), suggesting potential involvement of the viral oncogenes in the modulation of these pathways. Intracellular signaling components also showed weak positive correlations between AKT and mTOR (*r* = 0.45; *p* = 0.0104) and between EGFR and PI3K (*r* = 0.38; *p* = 0.0273), reinforcing the functional integration of these genes in regulating cellular processes related to tumor survival and proliferation (Figure 4, Table S3).

### Principal component analysis (PCA)

**Figure 5:**
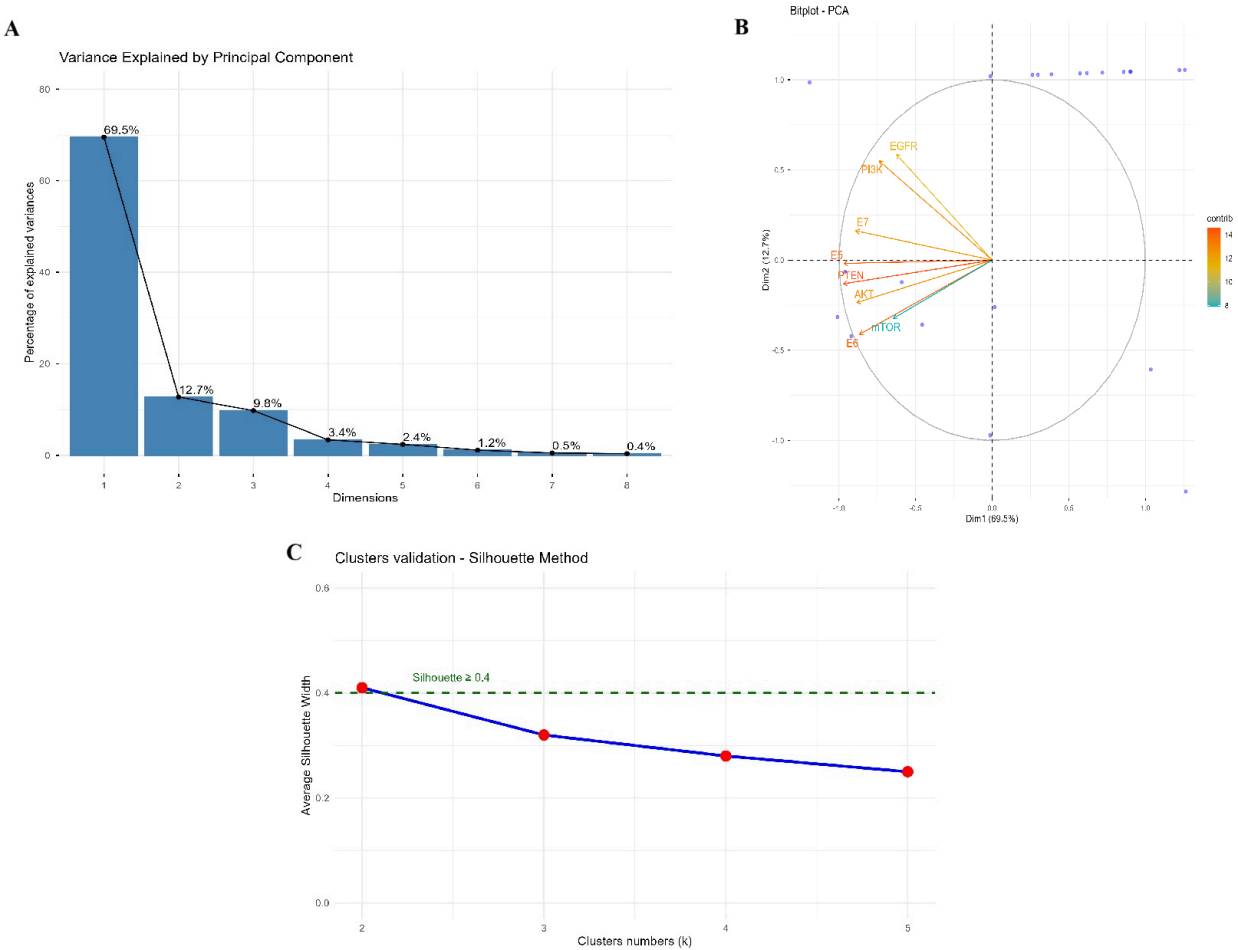
**PCA.** (**A**) Variance explained by each principal component (PC). Bars represent the individual variance explained by each PC. The first component (PC1) accounts for 69.5% of the total variance, whereas PC2 and PC3 explain 12.7% and 9.8%, respectively. (**B**) Principal component analysis (PCA) biplot showing the distribution of samples (blue points) and the contribution of genes (red arrows) across the first two PCs (PC1 and PC2). Longer arrows indicate greater contribution to component formation. The angle between arrows reflects gene–gene correlation: acute angles (< 90°) indicate a positive correlation, whereas obtuse angles (> 90°) indicate a negative correlation. The dashed circle represents the correlation circle (radius = 1). (**C**) Validation of the optimal number of clusters using the silhouette method. The average silhouette width was calculated for *k* = 2 to 5. The maximum value was observed at *k* = 2 (average silhouette = 0.41), exceeding the 0.4 threshold. The dashed green line indicates the cutoff for well-defined cluster separation (silhouette ≥ 0.4).

To investigate gene expression patterns associated with HPV infection, we analyzed exclusively HPV-positive samples (*n* = 37) using PCA and clustering analysis. First principal component (PC1) explained 69.5% of the variance and was predominantly driven by PTEN (–0.4118), E5 (–0.4105), E7 (–0.3783), and AKT (–0.3751) (Table S4). Silhouette validation confirmed a two-cluster structure (mean silhouette width = 0.41). The scree plot (Figure 5A) showed that PC1 accounted for 69.5% of the total variance, while PC2 and PC3 explained 12.7% and 9.8%, respectively, totaling 92.0% across the first three components. The biplot (Figure 5B) indicated that PTEN, E5, E7, and AKT made the largest contributions to PC1 (longest vectors), suggesting their central role in the molecular differentiation of samples. The distribution of samples suggested the presence of subgroups, which motivated clustering analysis. To determine the optimal number of clusters, silhouette analysis was applied (Figure 5C). The mean silhouette width was maximal at *k* = 2 (0.41), exceeding the 0.4 threshold recommended in the literature [35]. For *k* = 3, the value was 0.32, and for *k* ≥ 4, values were below 0.30. These results confirm that a two-cluster structure is statistically robust and adequately represents the molecular heterogeneity of HPV-positive samples (Figure S2).

### Gene expression heatmap analysis

**Figure 6:**
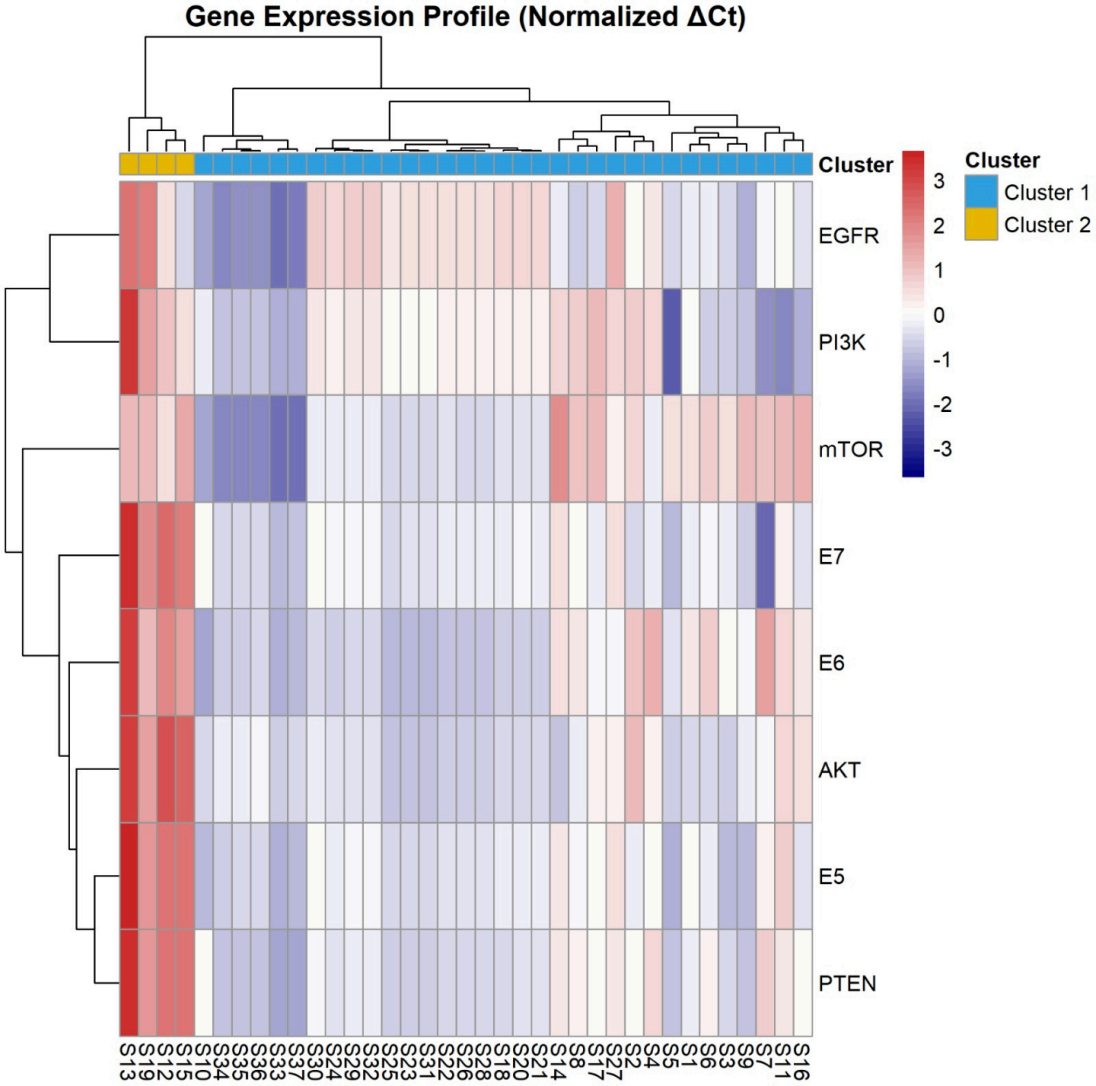
**Heatmap of gene expression in HPV-positive samples.** −ΔCt values were standardized (*z*-score scaling), and clustering was performed using Euclidean distance and Ward’s linkage method. Color intensity ranges from blue (low expression) to red (high expression), with white indicating intermediate expression levels. The top annotation bar indicates sample allocation into the two identified clusters (Cluster 1 in blue and Cluster 2 in yellow). A clear separation between clusters is observed: Cluster 1 is characterized by high expression of *PTEN*, *E5*, *AKT*, *E6*, and *E7*, and low expression of *mTOR*, *PI3K*, and *EGFR*, whereas Cluster 2 exhibits the opposite pattern. The average silhouette width of 0.41 further supports the quality of this separation.

To visualize gene expression profiles and validate the cluster structure identified by PCA, a heatmap was generated using standardized −ΔCt values (Figure 6). The heatmap revealed two distinct expression patterns, corresponding to the two clusters identified by the silhouette method (average silhouette width = 0.41). The clear visual separation between the two clusters in the heatmap supports the robustness of the clustering (silhouette = 0.41) and indicates that heterogeneity among HPV-positive samples is associated with distinct expression profiles within the PI3K/AKT/mTOR pathway.

## Discussion

To date, few studies have assessed the expression of viral oncogenes in breast cancer tissues, which serve as direct markers of HPV16 activity [36, 37]. A major challenge in this approach is the acquisition of high-quality RNA, particularly from FFPE tissues, which are more readily available for research than fresh specimens [38, 39]. In the present study, 92 FFPE breast tissue samples were analyzed. High-quality RNA was successfully isolated from only 48 of these samples, corroborating the challenges frequently reported in the literature for this type of analysis.

In our cohort of breast tumor samples, 77.08% were positive for HPV16. Among the HPV16-positive cases, 64.58% were classified as luminal A or luminal B molecular subtypes. The luminal subtype is the most prevalent among breast cancers and comprises a highly heterogeneous group in terms of prognosis and therapeutic response [40]. Luminal A breast cancer is characterized by low proliferative activity and a more favorable prognosis, whereas luminal B tumors exhibit higher proliferative rates, a more aggressive phenotype, and reduced post-recurrence survival [41, 42]. The high frequency of HPV16 may be associated with the widespread prevalence of this genotype in the general population, both in Brazil and globally. In Brazil, studies have shown that the prevalence of cervical HPV infection ranges from approximately 13.7% to 54.3%, with HPV16 representing the most prevalent high-risk genotype across all regions of the country [43, 44]. At the global level, high-risk HPV types represent a significant proportion of HPV-positive infections, accounting for 54.3%, although this prevalence may vary according to geographic region and the detection methods employed [45, 46]. Furthermore, other studies have reported consistent findings, demonstrating a higher prevalence of HPV16 in the luminal A and luminal B subtypes [47–49].

HPV oncogenes interact with multiple signaling pathways, thereby contributing to tumorigenesis [50]. As described in the literature, the E6 and E7 oncogenes interact with pRb and p53, respectively, facilitating cell cycle progression, whereas the E5 oncogene plays a key role in immune evasion [51, 52]. In contrast to the extensively studied E6 and E7 oncoproteins, which primarily deregulate cell cycle control and proliferation, the E5 oncogene is distinguished by its role in modulating growth factor receptor signaling and regulating the immune response [53]. The E5 oncogene interacts with the vacuolar H⁺-ATPase (V-ATPase), promoting EGFR activation by inhibiting EGF-induced endosomal acidification, thereby reducing receptor degradation. In addition, E5 downregulates the expression of human leukocyte antigen (HLA), contributing to immune evasion [50]. Previous studies have indicated that elevated levels of E5 can potentiate the activation of receptors such as the EGFR, triggering signaling cascades that promote cell proliferation and tumor progression [54–56].

The samples analyzed in this study exhibited higher relative expression of the E5 oncogene compared with E6 and E7. Furthermore, no significant difference in expression was observed between E6 and E7. Ren et al. [53] reported higher E5 expression in HPV-associated head and neck cancer samples compared to E6 and E7 levels. Similarly, Nascimento et al. [16] reported elevated E5 oncogene expression in HPV16-positive breast cancer samples; however, no comparison with E6 and E7 was performed. Collectively, these findings, together with the results presented here, suggest that the E5 oncogene may play a pivotal role in viral persistence in non-anogenital HPV-associated cancers, potentially contributing to the activation of non-canonical mechanisms involved in tumorigenesis.

E5 is known to disrupt the expression of major histocompatibility complex class I (MHC I), thereby deregulating the antigen presentation machinery and impairing the activation of CD8⁺ T lymphocytes [57]. Collectively, these interactions promote viral immune evasion and contribute to malignant cellular transformation. The expression of this oncogene in head and neck cancers has been associated with resistance to anti-PD-L1 immunotherapy, thereby impairing the generation of new T cells [58]. In this context, the data presented herein underscore the need for a more comprehensive investigation of E5 in breast cancer, particularly considering its overexpression and its potential impact on the response to immunotherapy used in the treatment of breast cancer, especially in the triple-negative subtype [59].

Breast carcinogenesis involves the dysregulation of cellular signaling pathways that drive proliferation and promote cell survival [60, 61]. Among the most frequently dysregulated pathways is the PI3K/AKT/mTOR signaling cascade, which is often upregulated in breast cancer and contributes to multiple processes essential for tumorigenesis [62, 63]. Uncontrolled activation of this pathway affects cellular metabolism, growth, and proliferation, as well as the regulation of apoptotic and angiogenic processes [64, 65]. In this study, we investigated for the first time the modulation of this pathway by HPV oncogenes in breast tumors, revealing key features that are discussed below.

Dysregulation of PI3K in breast cancer contributes to hyperactivation of this pathway, thereby increasing cell proliferation, promoting tumor progression, and driving therapeutic resistance [66]. Accordingly, PI3K inhibitors have been employed as a therapeutic strategy due to the high frequency of alterations in this pathway, including elevated expression and mutation rates in cancer [67]. Compounds such as GDC-0084 have demonstrated, in vitro, the ability to induce apoptosis and inhibit cell proliferation, suggesting potential therapeutic benefit [28]. Furthermore, a study by Liu et al. [68] demonstrated that HPV16-associated lung cancer cells exhibited higher expression levels of the viral oncogenes E6 and E7. Notably, increased expression of these genes was correlated with activation of the PI3K/AKT pathway, which regulates the activity of key transcription factors [68]. Additionally, a study in cervical carcinoma cells harboring the *E6* oncogene reported increased levels of phosphorylated PI3K, suggesting that HPV infection may influence the regulation of this protein [69]. However, this association warrants further investigation to confirm its significance and elucidate the underlying mechanisms.

AKT plays a central role in regulating cell proliferation, survival, and metabolism [70, 71]. In breast carcinoma, AKT can be activated by signaling pathways involving EGFR and PI3K and is frequently overexpressed in conjunction with PI3K, making it a key target for the development of novel therapeutic strategies [72, 73]. The results presented here revealed increased PI3K expression in the HPV16-positive group; however, no significant difference in AKT expression levels was observed between HPV16-positive and HPV16-negative breast cancer groups. These findings suggest that AKT expression may be regulated by alternative signaling pathways and post-transcriptional mechanisms beyond PI3K, potentially explaining the lack of significant differences at the mRNA level. In this context, our results also demonstrated a positive correlation between E6 and AKT, supporting the hypothesis that the absence of significant differences in AKT expression may be related to the low levels of E6 expression observed in this study. Notably, E6 has been shown to promote AKT phosphorylation, thereby enhancing the activity of eIF4E, a key regulator of the translation of genes involved in cell cycle progression, cell growth, and angiogenesis [74].

The mTOR plays a central role in regulating cellular metabolism, protein synthesis and degradation, survival, proliferation, and migration [75, 76]. Dysregulated oncogenes, hyperactivation of growth factor signaling via the PI3K/AKT and RAS/RAF pathways, elevated amino acid availability, and increased levels of pro-inflammatory cytokines collectively contribute to mTOR-driven tumorigenesis [77–79]. This gene is frequently dysregulated in breast cancer, either through gain-of-function mutations or increased activity of upstream signaling pathways [80]. In the present study, we observed a significant decrease in mTOR expression in the HPV16-positive breast cancer group (*p* < 0.001). Although previous studies have reported an association between HPV oncoproteins, particularly E6, and increased mTOR levels—and our correlation analysis also identified a positive association [81–83]—our findings indicate a reduction in mTOR expression in HPV16-positive samples. This discrepancy may be attributed to the relatively low levels of E6 expression observed in our cohort. Furthermore, it is possible that HPV suppresses mTOR expression as a strategy to evade immune surveillance, given that mTOR plays a central role in immune activation [76, 84].

Given the central role of this signaling cascade in breast carcinogenesis, inhibitors such as everolimus, in combination with exemestane, have been approved by the FDA for the treatment of metastatic, endocrine-resistant HR+/HER2− breast cancer with increased PI3K pathway activation [26]. Moreover, additional therapeutic strategies targeting other components of this pathway, including AKT inhibitors and mTORC1 inhibitors—the complex containing mTOR—are under development as potential treatments for breast cancer [26, 85–87]. Currently, these inhibitors are also being investigated for the treatment of HPV-positive malignancies, such as cervical cancer. A study by Lou et al. [88] (2026), using HPV-positive cervical cancer cell lines, demonstrated that inhibitors of the PI3K/AKT pathway, such as alpelisib and capivasertib, suppressed cellular proliferation, suggesting potential therapeutic benefit in advanced disease settings. Conversely, with regard to everolimus, a more favorable response has been observed in patients with HPV-negative head and neck cancer exhibiting higher mTOR expression [89]. Bossler et al. [84] demonstrated that HPV, particularly the E6 and E7 oncoproteins, can interact with and activate this signaling pathway in cervical cancer, representing a key mechanism in tumorigenesis. In this context, a comprehensive understanding of alterations in the effectors, activators, and inhibitors of this critical signaling cascade is essential to elucidate the role of HPV in breast cancer.

Cellular receptors such as EGFR activate PI3K, thereby positively regulating this signaling pathway. Upon activation, PI3K converts PIP2 into PIP3, leading to AKT phosphorylation and activation; in turn, active AKT promotes mTOR recruitment and activation [90]. Negative regulation of this pathway is mediated by the tumor suppressor PTEN, which dephosphorylates PIP3, thereby preventing AKT activation [91]. EGFR amplification is frequently observed in breast cancer, and increased EGFR copy number has been associated with poor prognosis, particularly in triple-negative breast cancer [92]. In cervical cancer, the E5 protein has been shown to promote cell proliferation by enhancing EGFR signaling [55]. In the present study, EGFR expression did not differ significantly between HPV16-positive and HPV16-negative breast cancer groups. However, correlation analysis revealed a positive association between EGFR and the viral oncogenes *E5* and *E7*, supporting the hypothesis that these genes may contribute to immune evasion and tumor proliferation by modulating the host immune response—particularly through interactions with MHC I molecules, as previously reported in cervical cancer [93, 94]. Consistent with our findings, a recent study by São Marcos et al. [95] in HPV-positive lung cancer cells associated the expression of E5, E6, and E7 with enhanced immune evasion and tumor progression.

PTEN is a PIP3-specific phosphatase that dephosphorylates this molecule, converting it back to PIP2, thereby acting as a negative regulator of the PI3K/AKT/mTOR pathway [96]. PTEN prevents PIP3 from recruiting AKT, thereby inhibiting this signaling cascade, which, when hyperactivated, contributes to tumorigenesis; PTEN thus functions as a critical tumor suppressor. PTEN expression levels may be modulated by the E6 oncoprotein through degradation of discs large homolog 1 (hDlg), a member of the membrane-associated guanylate kinase (MAGUK) protein family known to regulate PTEN activity [69, 97]. The present study demonstrated a trend toward reduced PTEN expression in HPV16-positive samples, consistent with previous reports showing that HPV16 may interfere with PTEN levels, leading to its downregulation, embora que em ambos os casos não ocorrou diferença estatistica significativa [97, 98]. In this context, Naderi et al. [99] (2025), in a study of patients with HPV-positive breast cancer, reported decreased PTEN expression and associated this downregulation with a potential mechanism contributing to breast tumor progression. Collectively, these findings suggest that this may represent one of the mechanisms through which HPV contributes to dysregulated cell growth in breast cancer. Given that PTEN is a key regulator of this pathway, its reduced expression, together with increased PI3K expression, may promote enhanced cellular proliferation.

Overall, our PCA and gene expression heatmap summarize and highlight important interactions among the targets analyzed in this study. The PCA revealed the formation of three distinct groups: the first, comprising HPV16 oncogenes (*E5*, *E6*, and *E7*) together with the human genes *PTEN* and *AKT* (PC1); the second, consisting of E6, EGFR, and PI3K (PC2); and the third, strongly dominated by mTOR (PC3). This finding supports the association between HPV oncogenes and the *PTEN* and *AKT* genes, which may be related to modulation of immune responses and cell proliferation, particularly given the reduced PTEN expression observed in the HPV-positive group. Regarding PC2, the opposing signals between EGFR/PI3K and E6 suggest that this component separates samples with high expression of the receptor–PI3K axis from those with higher expression of the viral oncogene *E6*, possibly reflecting the dual role of this oncogene at different stages of pathway activation. Finally, mTOR formed an independent cluster from the other genes, which may help explain its downregulation despite PI3K upregulation in the presence of HPV. These findings suggest a potential compensatory mechanism or dysregulation of the PI3K/AKT/mTOR axis in HPV-infected breast cancer cells. This phenomenon, together with the higher expression of the E5 oncogene compared with E6 and E7 observed in our study, may indicate a potential role of HPV-mediated signaling modulation in immune evasion mechanisms in breast tumors, as previously observed in other HPV-positive cancers [100, 101].

Furthermore, the low expression of these genes may also suggest that HPV modulates signaling pathways independent of PI3K/AKT/mTOR, such as the MAPK and JAK/STAT pathways [102, 103]. Given the use of pathway inhibitors in the treatment of breast cancer, these findings highlight the need for further studies with larger sample sizes to better elucidate the role of HPV oncogenes in modulating these signaling networks, which may ultimately impact treatment response in HPV-positive breast cancer patients.

### Conclusions

Our study revealed the expression of HPV16 oncogenes in 77.08% of the analyzed breast tumor samples, suggesting that HPV may represent a potential contributing factor in breast cancer. About viral oncogenes, this is the first study to comparatively assess the expression of E5, E6, and E7 in breast cancer. Our results demonstrated that the E5 oncogene exhibited higher relative expression compared with E6 and E7, underscoring the need for further investigations focused on E5 in breast tumors, given its critical role in immune evasion, sustained cellular proliferation, and the induction of therapeutic resistance. The PI3K/AKT/mTOR pathway, together with its upstream activator EGFR and downstream regulator PTEN, exhibited differential expression between HPV16-positive and HPV16-negative samples, characterized by activation of upstream signaling components and reduced expression of downstream elements in HPV-positive samples. Given that this pathway is a therapeutic target in breast cancer, our findings suggest that HPV status may influence treatment response. These findings reinforce the need for further investigations to elucidate the role of HPV in breast cancer, potentially enabling the future stratification of HPV-positive patients who may benefit from both preventive strategies, such as vaccination, and targeted therapies directed at genes within the PI3K pathway. However, this study employed advanced statistical approaches, including PCA and multiple comparison procedures, in a relatively small sample size, which may limit the robustness of the reported *p*-values. Therefore, studies with larger sample sizes are required to validate the hypothesis proposed in this study.

## Abbreviations

CI: confidence interval
EGFR: epidermal growth factor receptor
FDA: Food and Drug Administration
FFPE: formalin-fixed, paraffin-embedded
HPV: human papillomavirus
MHC I: major histocompatibility complex class I
mTOR: mammalian target of rapamycin
ORs: odds ratios
PC1: first principal component
PCA: principal component analysis
PIP2: phosphatidylinositol 4,5-bisphosphate
PIP3: phosphatidylinositol (3,4,5)-trisphosphate
PTEN: phosphatase and tensin homolog
RT-qPCR: real-time quantitative PCR

## Supplementary materials

The supplementary materials for this article are available at: https://www.explorationpub.com/uploads/Article/file/1002391_sup_1.pdf.

## Declarations

### Availability of data and materials

The data are available and can be accessed by contacting the corresponding author.

### Copyright

© The Author(s) 2026.
