# Supplementary material for: Transcriptional modulation of the PI3K/AKT/mTOR signaling pathway mediated by HPV16 E5, E6, and E7 oncogene expression in breast cancer
Source: Explor Target Antitumor Ther. 2026 Aug 4;7:1002391. doi: 10.37349/etat.2026.1002391 (PMC13439120; doi:10.37349/etat.2026.1002391)
Supplement: Supplementary file 1 [file 1002391_sup_1.pdf]

Table S1. Sample characterization including clinical data, RNA quality from breast tumors, and HPV status determined by quantification of E5, E6, and E7 oncogenes.

ID	Age	Molecular classification	Histological type	Quality	RNA quantification (ng/ul)	260/280 ratio	Status HPV
21	46	HER-2	Invasive Ductal	no	137.2	1.64	-
22	49	Luminal A	Other types	no	137.2	1.71	-
25	35	Luminal A	Other types	no	179.3	1.5	-
26	80	Her-2	Other types	no	204.2	1.57	-
27	80	Her-2	Invasive Ductal	no	-	-	-
28	85	Luminal A	Other types	yes	227	1.95	negative
29	66	-	Invasive Ductal	yes	453.6	2.1	negative
30	69	Luminal A	Other types	no	179	1.14	-
31	75	Luminal A	Ductal in situ	yes	294.1	1.97	negative
32	66	-	Not-identified	yes	486.8	2.19	negative
33	46	Luminal B	Not-identified	yes	244.3	1.92	negative
34	68	luminal B	Ductal in situ	yes	259.5	2.1	positive
35	36	luminal B	Invasive Ductal	yes	167.3	1.87	Positive
36	69	luminal A	Invasive Ductal	yes	219	1.95	Positive
37	59	luminal A	Ductal in situ	yes	225.3	1.88	Positive
38	52	Luminal A	Invasive Ductal	yes	308.8	2	Negative
39	72	luminal B	Invasive Ductal	yes	186.2	1.9	Positive
40	36	Her- 2	invasive Ductal	yes	413.9	2.14	Negative
41	47	Triple negative	invasive Ductal	yes	373.3	2.14	Positive
42	61	Luminal A	invasive Ductal	yes	157.8	1.91	positive
43	72	luminal A	Ductal in situ	no	357.6	1.11	-
44	50	Luminal A	Invasive Ductal	yes	292.8	2.02	positive
45	56	Luminal B	invasive Ductal	yes	170	1.94	negative
46	62	luminal B	Other types	yes	570.2	2.13	negative
47	39	Luminal B	invasive Ductal	yes	307.6	2.1	positive
48	61	luminal B	invasive Ductal	yes	342.6	2.15	negative
51	78	luminal A	invasive Ductal	yes	226.1	2.02	positive
52	72	Luminal A	Invasive Ductal	yes	339.6	2.13	positive
54	41	Luminal B	Not identified	yes	159.9	2.07	positive
55	53	-	Not identified	yes	239.7	2.04	positive
56	62	Luminal A	invasive Ductal	yes	287	2.09	negative
57	65	Luminal A	invasive Lobular	no	302.7	2.39	-
58	41	Triple negative	Invasive Ductal	no	12.4	1.84	-
59	64	Luminal A	Other types	no	75.6	1.88	-
60	57	Triple negative	Invasive Ductal	yes	319.8	2.11	positive
61	79	Luminal A	Invasive Ductal	yes	777.2	2.12	positive
62	61	Luminal A	Invasive Ductal	yes	232.2	1.94	positive
63	70	Her-2	Other types	no	140.1	1.34	-
66	29	Luminal A	Other types	no	34.3	2.43	-
68	74	Lminal A	Ductal in situ	no	24.7	2.07	-
71	48	Luminal B	invasive Ductal	no	31.8	2.1	-
72	57	Luminal B híbrido	invasive Ductal	no	43.8	2.05	-

74	66	Luminal B	Other types	no	250.7	2.69	-
75	47	Luminal A	Other types	no	204.9	1.44	-
76	72	LUMINAL A	invasive Lobular	no	200.8	1.27	-
77	61	Luminal A	Ductal in situ	no	195.2	1.33	-
78	59	Luminal A	Invasive Ductal	no	269.8	2.42	-
79	57	Her-2	Ductal in situ	no	26.9	2.03	-
80	72	Luminal A	Other types	yes	306	2.02	positive
81	57	Luminal B	Invasive Ductal	no	399.1	2.54	-
82	56	Triple negative	Invasive Ductal	no	42.3	1.85	-
83	51	Luminal A	Invasive Ductal	no	190.4	1.55	-
84	33	Luminal A	Invasive Ductal	no	45.6	1.86	-
85	61	Luminal A	Ductal in situ	no	398.4	2.58	-
86	62	Luminal A	Invasive Ductal	no	437.3	1.45	-
88	47	Triple negative	Invasive Ductal	yes	169.2	1.07	positive
89	74	Luminal B	Invasive Ductal	no	76.3	1.8	-
90	50	Triple negative	Invasive Ductal	yes	290.4	2.09	positive
91	74	Luminal B	Invasive Ductal	no	478.6	2.51	-
92	58	Luminal B	Invasive Ductal	no	128.8	0.92	-
94	49	Luminal A	Invasive Ductal	no	167	1.08	-
95	49	Luminal B	Ductal in situ	yes	151.2	1.87	positive
97	74	Luminal A	Invasive Ductal	no	428.5	2.32	-
99	51	Luminal A	Invasive Ductal	yes	274.9	1.99	positive
100	81	Luminal A	Invasive Ductal	yes	268.2	1.99	positive
101	52	Her-2	Invasive Ductal	no	24.9	2.06	-
102	69	Luminal B	Invasive Ductal	no	21.5	2.26	-
103	74	luminal B	Invasive Ductal	no	271.2	1.05	-
104	29	Luminal A	Other types	no	55.3	2.15	-
105	55	Luminal B	Invasive Ductal	yes	446	2.17	positive
106	-	-	Not-identified	yes	396.4	2.09	positive
107	47	Luminal A	Invasive Ductal	yes	328.9	2.02	positive
108	-	-	Not-identified	yes	842.2	2.22	positive
110	51	Luminal A	Invasive Ductal	yes	271.1	2.01	positive
112	59	Luminal B	Invasive Ductal	yes	385.9	2.11	positive
113	59	Luminal A	Invasive Ductal	no	268.9	1.25	-
114	49	Luminal A	Ductal in situ	yes	211.8	1.96	positive
115	64	Luminal B	Invasive Ductal	no	5.8	1.85	-
116	63	Luminal A	Invasive Ductal	yes	224.9	1.97	positive
117	57	Luminal B	Invasive Ductal	yes	257.2	2.02	positive
121	74	Luminal A	Other types	no	205.9	1.19	-
122	37	Luminal B	Invasive Ductal	no	47.4	1.54	-
124	66	Luminal B	Other types	no	45.5	1.33	-
125	60	Luminal B	Invasive Ductal	no	45	1.05	-
127	69	Her-2	Invasive Ductal	yes	156.2	1.99	positive
130	54	Luminal B	Ductal in situ	no	-	-	-
133	66	Luminal B	Invasive Ductal	yes	343.1	1.83	positive
135	72	Luminal A	Ductal in situ	yes	137.8	1.84	positive

136	70	Luminal B	Invasive Ductal	yes	178.5	2	positive
137	39	Luminal B	Invasive Ductal	yes	477.3	2.2	positive
138	68	Luminal A	Other types	no	12.4	1.51	-
141	83	Luminal B	Invasive Ductal	yes	181.3	1.93	positive

Table S2. Primers used for gene expression analysis of PI3K, AKT, mTOR, PTEN, E5, E6, E7, and EGFR via RT-qPCR. The genes EEF1A1 and GAPDH were used as reference genes for normalization of gene expression.

ID	Sense	Sequence	Amplicon size	Efficiency	Reference
E5_HPV16	F	ACAACATTACTGGCGTGCT	140	93.07%	Ren et al., 2020
E5_HPV16	R	GAGGCTGCTGTTATCCACAATA	140	93.07%	Ren et al., 2020
E6_HPV16	F	TCAGGACCCACAGGAGCG	109	108.20%	Ren et al., 2020
E6_HPV16	R	CCTCACGTCGCAGTAACTGTTG	109	108.20%	Ren et al., 2020
E7_HPV16	F	CCGGACAGAGCCCATTACAA	92	109.67%	Ren et al., 2020
E7_HPV16	R	CGAATGTCTACGTGTGTGCTTTG	92	109.67%	Ren et al., 2020
PI3K	F	GGGGATGATTTACGGCAAGATA	144	100.08%	Zhang et al., 2019
PI3K	R	CACCACCTCAATAAGTCCCACA	144	100.08%	Zhang et al., 2019
AKT	F	GCAGCACGTGTACGAGAAGA	67	102.65%	Zhang et al., 2019
AKT	R	GGTGTCAGTCTCCGACGTG	67	102.65%	Zhang et al., 2019
mTOR	F	CGCTACTGTGTCTTGGCATC	154	103.09%	Tong et al., 2021
mTOR	R	GGTTCATGCTGCTTAGTCGG	154	103.09%	Tong et al., 2021
PTEN	F	CAGTAGAGGAGCCGTCAAATC	108	106.28%	Qin et al., 2020
PTEN	R	CAGAGTCAGTGGTGTCAGAAT ATC	108	106.28%	Qin et al., 2020
EGFR	F	AACACCCTGGTCTGGAAGTAC G	106	94.92%	(NM_005228, HP208404, OriG ene Technologies, Inc., Beijing, China)
le EGFR	R	TCGTTGGACAGCCTTCAAGAC C	106	94.92%	(NM_005228, HP208404, OriG ene Technologies, Inc., Beijing, China)
GAPDH	F	GAAGGCTGGGGCTCATTTG	154	105.81%	Leitao MdCG et al., 2014
GAPDH	R	TAAGCAGTTGGTGGTGCAGG	154	105.81%	Leitao MdCG et al., 2014
EEF1A1	F	GTTGCGGTGGGTGTCATCA	123	94.54%	Leitao MdCG et al., 2014

EEF1A1	R	GAGTGGGGTGGCAGGTATT	123	94.54%	Leitao MdCG et al., 2014

The efficiency was calculated using Cq values from cDNA samples of MDA cell lines (for the genes PI3K, AKT, mTOR, EGFR, and PTEN) and SiHa cells (for HPV oncogenes). The calculation was performed in Excel using the formula: Efficiency = −1 + 10^(−1/slope) × 100.

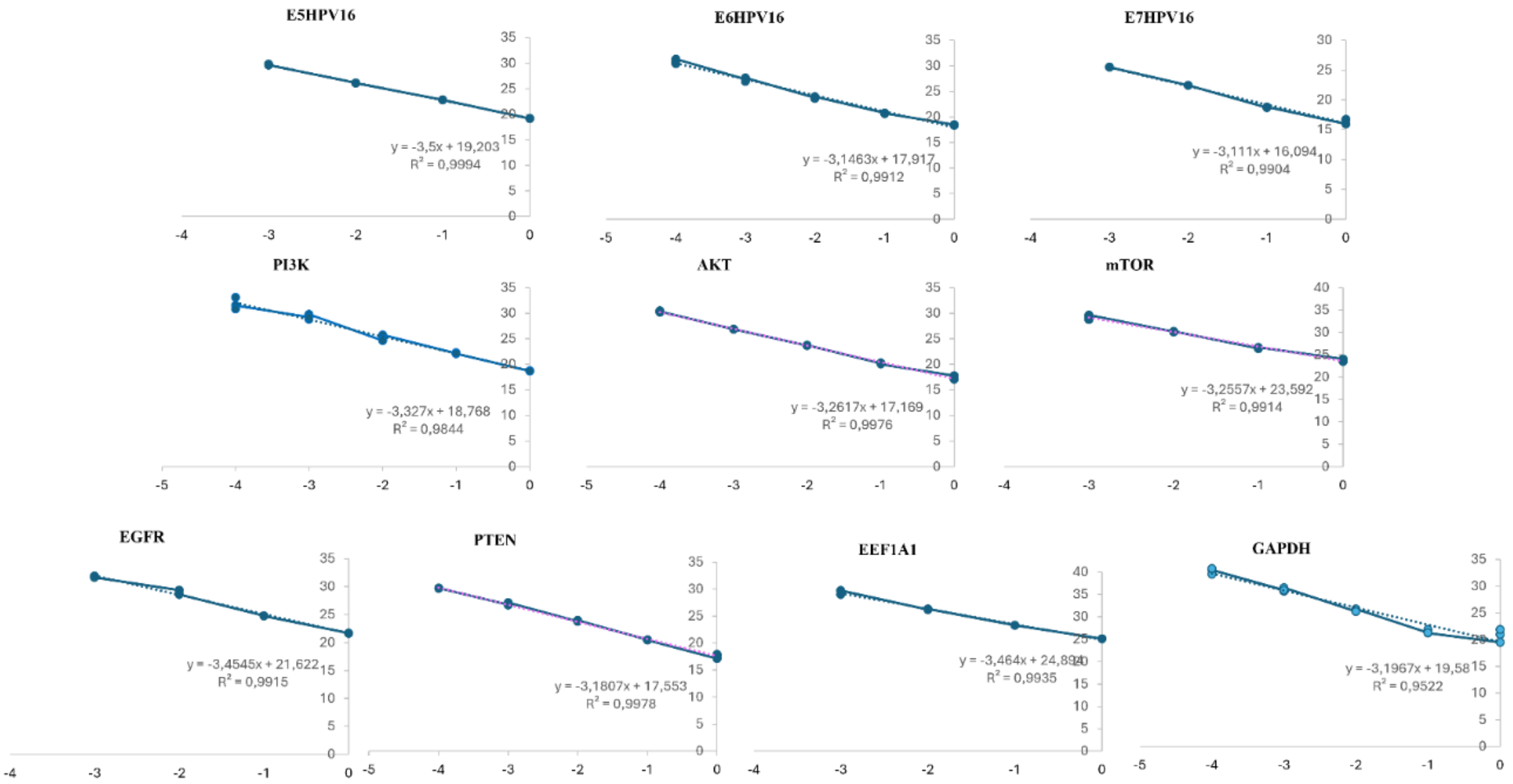

Figure S1. Efficiency curve of the primers described in Table S2.

Table S3. Spearman correlation performed in GraphPad Prism between the HPV genes E5, E6, and E7 and the human genes EGFR, PI3K, AKT, mTOR, and PTEN.

	E5	E6	E7	EGFR	PI3K	AKT	mTOR	PTEN
**E5**		0.003978	0.055558	0.073498	0.096009	0.133985	0.282457	0.066094
**E6**	0.003978		0.995894	0.588734	0.311336	0.000221	0.014593	0.053191
**E7**	0.055558	0.995894		0.018615	0.019464	0.254839	0.130152	0.812399
**EGFR**	0.073498	0.588734	0.018615		0.027311	0.312177	0.811066	0.774638
**PI3K**	0.096009	0.311336	0.019464	0.027311		0.125653	0.429134	0.094664
**AKT**	0.133985	0.000221	0.254839	0.312177	0.125653		0.01042	0.019034
**mTOR**	0.282457	0.014593	0.130152	0.811066	0.429134	0.01042		0.445841
**PTEN**	0.066094	0.053191	0.812399	0.774638	0.094664	0.019034	0.445841	

Table S4. Gene loadings for the first three principal components (PC1, PC2, and PC3) obtained from PCA of −ΔCt values in HPV-positive samples (n = X). Loadings in bold indicate absolute values > 0.3, considered most relevant for component composition. The percentage contribution to PC1 was calculated as (∣loading∣ / Σ∣loadings∣) × 100. PC1 explains 69.5% of the total variance, whereas PC2 and PC3 account for 12.7% and 9.8%, respectively.

Gene	PC1	PC2	PC3	Contribuição PC1 (%)	Interpretação
E5	**-0.4105**	-0.0181	-0.1458	14.7	Viral oncogene– activates of EGFR.
PTEN	**-0.4118**	-0.1302	-0.0698	14.7	Tumor supressor - degrades by HPV
E7	**-0.3783**	0.1612	**-0.2961**	13.5	Viral oncogene inactivates pRb
AKT	**-0.3751**	-0.2333	**-0.3460**	13.4	PI3K/AKT pathway – central protein.
E6	**-0.3672**	**-0.4074**	0.0912	13.1	Viral oncogene - degrades p53
PI3K	-0.3132	**0.5450**	-0.0853	11.2	PI3K/AKT pathway - activates by EGFR
mTOR	-0.2736	-0.3197	**0.7290**	9.8	Pathway effector regulates proliferation.
EGFR	-0.2652	**0.5806**	**0.4684**	9.5	Receptor tyrosine kinase activated by E5

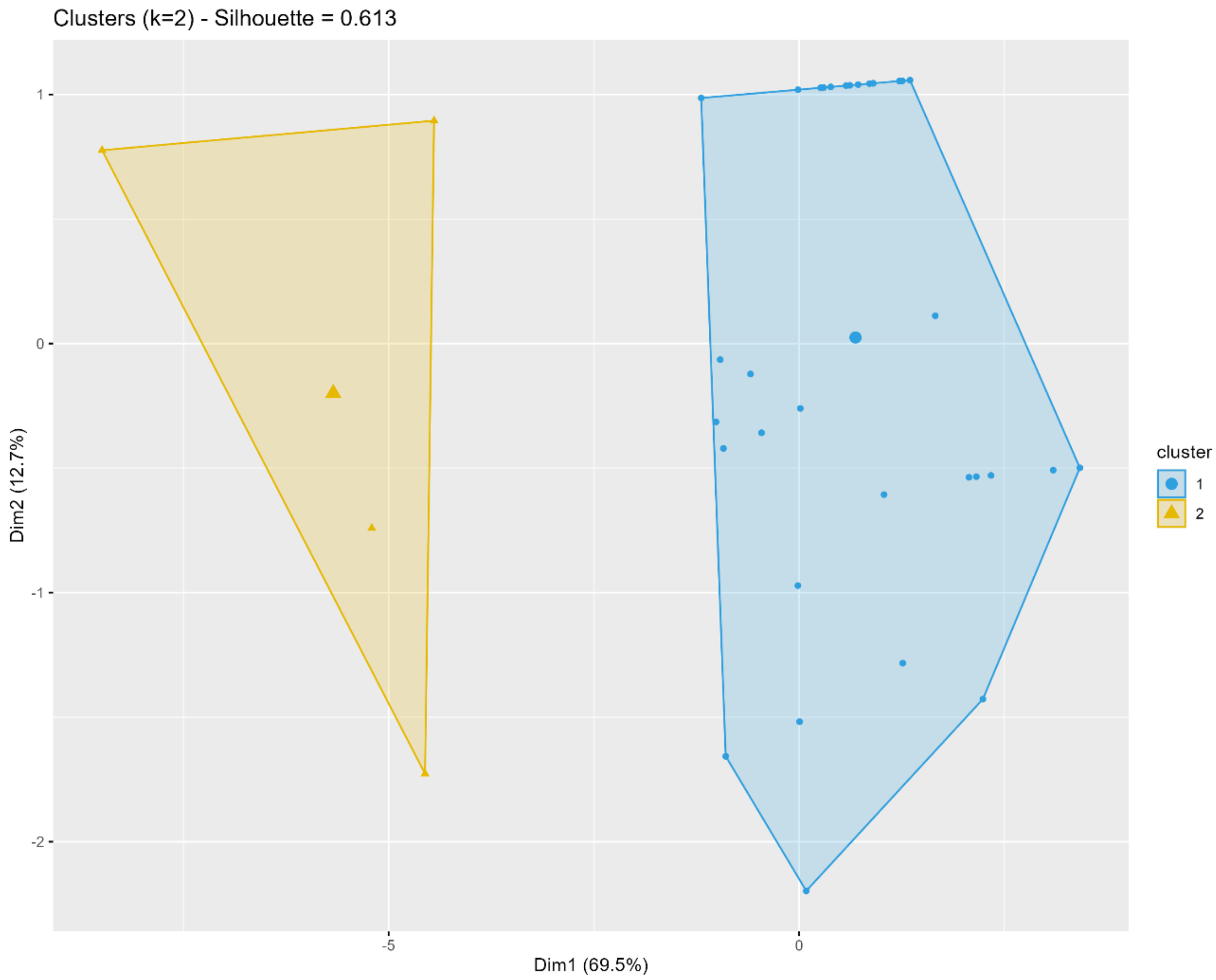

Figure S2. Visualization of clusters generated by the k-means algorithm (k = 2). Each point represents an HPV-positive sample, colored according to its cluster assignment (Cluster 1: blue; Cluster 2: yellow). Ellipses denote the 95% confidence interval for each cluster.
